# AEBP1 Promotes Glioblastoma Progression and Activates the Classical NF-*κ*B Pathway

**DOI:** 10.1155/2020/8890452

**Published:** 2020-11-06

**Authors:** Kai Guo, Lei Song, Jianyong Chang, Peicheng Cao, Qi Liu

**Affiliations:** Department of Neurosurgery, Weifang People's Hospital, Weifang, Shandong, China 261000

## Abstract

**Objective:**

Our study was aimed at investigating the mechanistic consequences of the upregulation of *adipocyte enhancer-binding protein 1* (*AEBP1*) in glioblastoma (GBM).

**Methods:**

The expression of *AEBP1* in GBM was assessed by bioinformatics analysis and qRT-PCR; the effects of *AEBP1* on GBM cell proliferation, migration, invasion, and tumor growth in vitro and in vivo were detected by a CCK-8 assay, colony formation assay, scratch assay, Transwell assay, and subcutaneous tumor formation, respectively. The activation of related signaling pathways was monitored using western blot.

**Results:**

Tumor-related databases and bioinformatics analysis revealed that *AEBP1* was highly expressed in GBM and indicated poor outcome of patients; its high expression that was also confirmed in GBM tissues and cell lines was closely related to the tumor size. The results of in vitro experiments showed that *AEBP1* could significantly promote GBM cell proliferation, migration, and invasion; in vivo experiments suggested that *AEBP1* could contribute to the growth of GBM tumors. *AEBP1* could upregulate the level of *IκBα* phosphorylation, decrease *IκBα* expression, activate the NF-*κ*B signaling pathway, and promote the expression of downstream oncogenes.

**Conclusion:**

Upregulated *AEBP1* in GBM promotes GBM cell proliferation, migration, and invasion and facilitates tumor growth in vivo by activating the classical NF-*κ*B pathway.

## 1. Introduction

Glioblastoma (GBM) is a common primary malignant brain tumor in adults. Known as a very aggressive brain cancer, it is one of the most lethal malignancies in humans [1, 2]. At present, the prognosis of patients with GBM remains very poor, with a median survival time of 15 months [3, 4]. Therefore, an in-depth investigation of the molecular mechanisms underlying the development of GBM is conducive to advances in GBM diagnosis and treatment.

*Adipocyte enhancer-binding protein 1* (*AEBP1*) is a transcriptional repressor with carboxypeptidase (CP) activity. The AEBP1 protein could positively regulate the activity of MAP kinase (MAPK) in adipocytes, thus stimulating adipocyte proliferation and reducing adipocyte differentiation; the proteins can also positively regulate NF-*κ*B activity in macrophages by promoting I-kappa-B-alpha (NFKBIA) phosphorylation and subsequent degradation, thereby enhancing the inflammatory responsiveness of macrophages [5]. *AEBP1* is involved in the progression of a variety of diseases, such as abdominal aortic aneurysm [6], nonalcoholic steatohepatitis [7], Ehlers-Danlos syndrome [8], and Alzheimer's disease [9]. Studies have shown that *AEBP1* is highly expressed in a variety of malignant tumors (such as breast cancer, glioblastoma, bladder cancer, gastric cancer, colorectal cancer, ovarian cancer, and skin cancer) [5]. *AEBP1* can promote proliferation, metastasis, angiogenesis, and inflammation and suppress apoptosis both in vitro and in vivo; therefore, it acts as an oncogene to promote tumor progression [5]. For example, upregulation of *AEBP1* in colon cancer accelerates the progression of colon cancer by promoting angiogenesis [10]. *AEBP1* is highly expressed in both primary and recurrent childhood acute lymphoblastic leukemia. Silencing of *AEBP1* can inhibit Jurkat cell proliferation through a p53-dependent pathway and promote apoptosis [11]. The expression of *AEBP1* is significantly increased in human gastric cancer and correlates with poor patient outcome; *AEBP1* can significantly facilitate the proliferation, migration, invasion, and epithelial-mesenchymal transition of gastric cancer cells [12].

*AEBP1* has been reported to enhance glioma cell survival [13], while silencing *AEBP1* causes caspase-dependent death of GBM cells; therefore, *AEBP1* is a potential oncogenic driver in glioma [14]. To date, studies on the role of *AEBP1* in GBM have mostly focused on apoptosis. However, since GBM is very aggressive, it is equally essential to study in depth the effects of *AEBP1* on GBM proliferation, migration, and invasion. Therefore, this study further investigates the consequences of the upregulation of *AEBP1* in GBM and its clinical significance, by exploring the effects of *AEBP1* expression on glioma proliferation, migration, invasion in vitro, and tumor growth in vivo as well as the underlying mechanisms.

## 2. Materials and Methods

### 2.1. Bioinformatics Analysis

The UCSC Xena database (https://xena.ucsc.edu) [15] was used to analyze the differential expression of *AEBP1* in GBM tissues versus normal brain tissues. The effect of *AEBP1* expression on disease prognosis was also analyzed based on TCGA database (http://cancer.genome.nih.gov). *P* < 0.05 was considered statistically significant.

### 2.2. Sample Acquisition

A total of 51 cases of GBM tissue and corresponding 51 cases of normal brain tissue adjacent to cancer were included in this study. All GBM tissues were confirmed by histopathology, and none of the adjacent tissues contained cancerous components (see Supplementary File 1). All tissues were obtained from patients undergoing surgery at our hospital, and informed consent was obtained from all the patients. None of the selected patients received radiotherapy or chemotherapy before surgery. All the patients were retrospectively enrolled in the current study. This study has been approved by the hospital ethics committee.

### 2.3. Cell Culture

Human GBM cell lines U251, U87, A172, and LN229 and normal human astrocytes (NHAs) were purchased from American Type Culture Collection (ATCC, USA). DMEM (Gibco, USA) supplemented with 10% fetal bovine serum (Gibco) was used to culture the cells. All cell lines were maintained in a humidified atmosphere containing 5% CO_2_ at 37°C.

### 2.4. Cell Transfection with a Plasmid

*AEBP1* siRNAs were purchased from Sigma-Aldrich (USA); its target sequence was TGGACACCAGGAGGACTACCCGGTTCACAGGCGTCATCACCCAGGGCAGAGACTCCAGCATCCATGACGATTTTGTGACCACCTTCTTCGTGGGCTTCAGCAATGACAGCCAGACATGGGTGATGTACACCAACGGCTATGAGGAAATGACCTTTCATGGGAACGTGGACAAGGACACACCCGTGCTGAGTGAGCTCCCAGAGCCGGTGGTGGCTCGTTTCATCCGCATCTACCCACTCACCTGGAATGGCAGCCTGTGCATGCGCCTGGAGGTGCTGGGGTGCTCTGTGGCCCCTGTCTACAGCTACTACGCACAGAATGAGGTGGTGGCCACCGATGACCTGGATTTCCGGCACCACAGCTACAAGGACATGCGCCAGCTCATGAAGGTGGTGAACGAGGAGTG. The *AEBP1* expression plasmid was constructed by inserting the *AEBP1* coding region into the PCDNA3.1 vector. Cell transfection was performed using the Lipofectamine 2000 reagent (Invitrogen, USA). Transfection efficiency was assessed using qRT-PCR and western blot.

### 2.5. Real-Time qRT-PCR

The relative mRNA expression of *AEBP1* was evaluated using qRT-PCR. Total RNA was extracted from GBM tissues and cell lines using the TRIzol reagent (Invitrogen). RNA was reversely transcribed into cDNA using a reverse transcription kit (Takara, Japan). PCR reactions were performed with the RNA-Direct SYBR Green Real-Time PCR Master Mix (Toyobo, Japan) and Roche LightCycler 480 Real-Time PCR System (Applied Biosystems, USA). The relative expression of genes was calculated by the 2^-*ΔΔ*Ct^ method. The primers used for the experiment were as follows: *AEBP1*, forward: 5′-ACCCACACTGGACTACAATGA-3′ and reverse: 5′-GTTGGGGATCACGTAACCATC-3′, and *GAPDH* used as the internal reference, forward: 5′-TATGATGATATCAAGAGGGTAGT-3′ and reverse: 5′-TGTATCCAAACTCATTGTCATAC-3′.

### 2.6. Western Blot

Protein expressions of *AEBP1*, phosphorylated *IκBα*, *IκBα*, phosphorylated *NF-κB p65*, *NF-κB p65*, *Cyclin D1* (*CCND1*), *MYC Proto-Oncogene* (*c-Myc*), *Matrix Metallopeptidase 9* (*MMP9*), and *Snail Family Transcriptional Repressor 2* (*Slug*) were detected by western blot. GBM cells were lysed with RIPA buffer containing protease inhibitors. Total proteins were separated by electrophoresis in 10% SDS-PAGE and subsequently transferred to PVDF membranes. The membranes were then incubated with primary antibodies overnight at 4°C, followed by an incubation with secondary antibodies for 1 h at room temperature. Immunoreactive bands were revealed by chemiluminescence, and relative expression of the target protein was normalized to that of *GAPDH* used as an internal reference. The primary antibodies used in this study were purchased from Abcam (USA): anti-AEBP1 (ab168355), anti-I*κ*B*α* (ab7217), anti-p-I*κ*B*α* (ab133462), anti-NF-*κ*B p65 (ab16502), anti-p-NF-*κ*B p65 (ab86299), anti-CCND1 (ab16663), anti-c-Myc (ab39688), anti-MMP9 (ab38898), anti-Slug (ab27568), and anti-GAPDH (ab8245).

### 2.7. Cell Proliferation Assay

Cell proliferation was assessed using the CCK8 assay and colony formation assay. For the CCK8 assay, after cell transfection, 1 × 10^3^ GBM cells were seeded on 96-well plates. After 24, 48, and 72 hours of incubation, CCK-8 solution (APExBIO, USA) was added to each well for further incubation of the cells and the absorbance at 450 nm was measured with a spectrophotometer. For the colony formation assay, after cell transfection, cells were seeded in 6-well plates at a density of 500 cells/well and cultured for 2 weeks. After the colonies had formed, they were fixed with 4% paraformaldehyde, stained with 0.1% crystal violet, and counted as part of clonogenic assays.

### 2.8. Cell Migration and Invasion Assays

Cell migration was assessed by a scratch assay. GBM cells were seeded on 6-well plates. 48 hours after transfection, 3 parallel scratch wounds were introduced in the cell monolayer with a pipette tip. The cells were gently rinsed twice with PBS, followed by incubation with DMEM containing 1% fetal bovine serum. Pictures of the scratch were taken at 0 and 24 hours. Cell invasion was assessed by a Transwell assay. GBM cells were seeded in the upper chamber of the cell culture insert coated with Matrixgel (BD Biosciences, USA). The medium without or with 10% FBS was added to the upper and lower chambers, respectively, and the culture was extended for another 24 hours. Then, the cells in the upper chamber that have not penetrated the membrane are wiped off with a cotton swab. The cells in the lower layer that passed through the membrane were fixed with methanol and stained with 0.1% crystal violet. The cells were counted under an inverted microscope.

### 2.9. Subcutaneous Tumor Formation

Five-week-old male BALB/C mice were purchased from Changzhou Cavens Laboratory Animal Co., Ltd. U251 cells (1 × 10^7^) with stable overexpression of *AEBP1* or U87 cells stably silencing *AEBP1* after lentiviral infection were injected subcutaneously into the left flank. At the end of the experiment, mice were euthanized by cervical dislocation. This study conformed to the *Guide for the Care and Use of Laboratory Animals* of the National Institutes of Health. The protocol was approved by the hospital ethics committee.

### 2.10. NF-*κ*B Activity Measurement

A luciferase reporter-based experiment was used to assess the transcriptional activity of NF-*κ*B in cells. Cells were cotransfected with a p-NF-*κ*B-Luc luciferase reporter gene plasmid (Beyotime, China) and a p-RL-TK Renilla fluorescent plasmid and collected after 24 hours in culture. The fluorescence intensity was detected using a dual-luciferase reporter gene detection kit (Promega, USA), and the firefly luciferase fluorescence intensity/Renilla luciferase fluorescence intensity ratio was used as a read out of the relative transcription activity of NF-*κ*B.

### 2.11. Statistical Analysis

All experiments were performed at least three times. All experimental data are presented as mean ± standard deviation. Data analysis was carried out using SPSS 20.0 software (USA). Differences were analyzed using *t*-tests or one-way variances. *P* value < 0.05 was considered statistically significant for differences.

## 3. Results

### 3.1. Bioinformatics Analysis of *AEBP1* Expression in GBM

**Figure 1:**
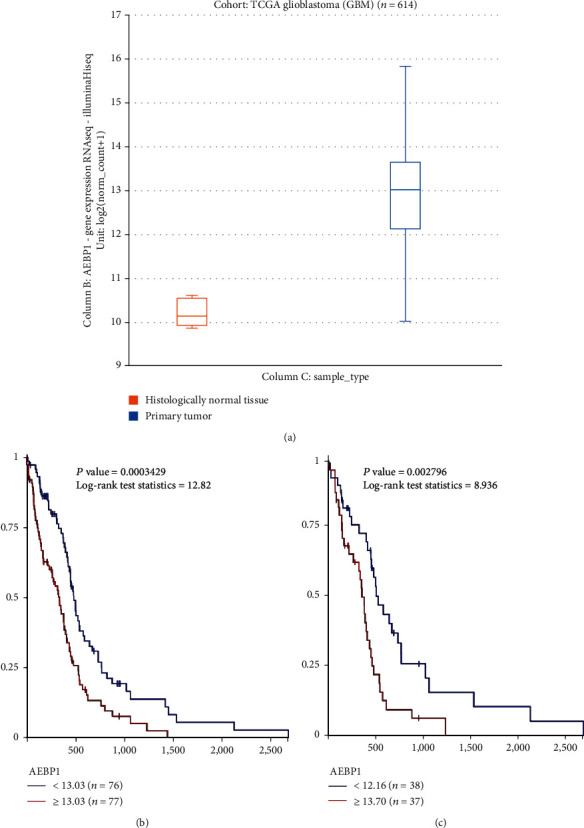
Bioinformatics analysis of *AEBP1* expression in GBM: (a) *AEBP1* expression in GBM analyzed through the Xena database; (b, c) effect of *AEBP1* expression on the overall survival of GBM patients analyzed through TCGA database.

According to the Xena database, *AEBP1* was overexpressed in GBM tissues compared with adjacent histologically normal brain tissue; this difference is statistically significant (Figure 1(a)). Moreover, TCGA database documented that GBM patients with high *AEBP1* expression had a shorter overall survival (Figures 1(b) and 1(c)).

### 3.2. Expression of *AEBP1* in GBM Tissues and Cells and Underlying Clinical Significance

**Figure 2:**
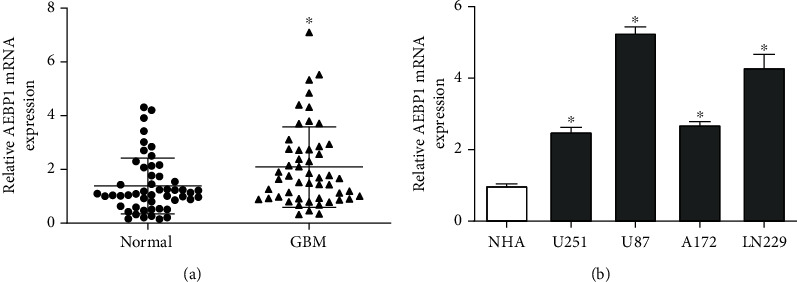
Expression of *AEBP1* in (a) GBM tissues and (b) cell lines. ^∗^*P* < 0.05.

According to our qRT-PCR results for 51 pairs of GBM tissues and adjacent normal tissues, *AEBP1* showed significantly higher mRNA expression in tumor tissues (Figure 2(a)). *AEBP1* mRNA expression was also significantly higher in GBM cell lines U251, U87, A172, and LN229 than in normal human astrocytes (NHAs) (Figure 2(b)). Moreover, the expression of *AEBP1* was found to be closely related to tumor size based on the clinical data of the patients (Table 1). These results indicated that *AEBP1* was highly expressed in GBM tissues and cell lines, supporting its contribution to GBM disease progression.

### 3.3. *AEBP1* Overexpression and Silencing in GBM Cells

**Figure 3:**
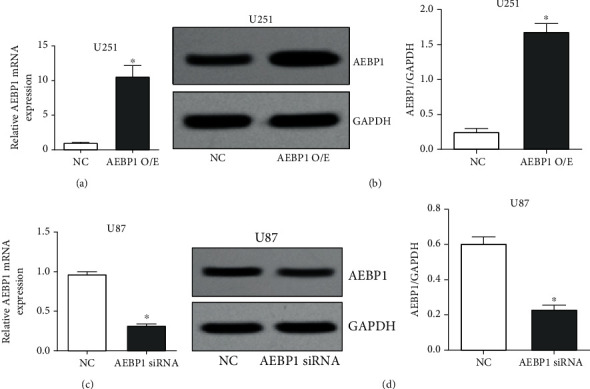
Validation of *AEBP1* overexpression and silencing in GBM cells. Transfection of the *AEBP1* overexpression plasmid in U251 cells and *AEBP1* siRNA in U87 cells: (a, c) detection of *AEBP1* mRNA expression by qRT-PCR; (b, d) detection of *AEBP1* protein expression by western blot. *N* = 3, ^∗^*P* < 0.05.

The *AEBP1* overexpression plasmid was transfected in U251 cells, the cell line with the lowest baseline *AEBP1* expression. Both qRT-PCR and western blot results showed that *AEBP1* mRNA and protein levels were both significantly increased after transfection (Figures 3(a) and 3(b)). Conversely, *AEBP1* siRNA was transfected in U87 cells, the cell line with the highest *AEBP1* expression. The results from qRT-PCR and western blot analyses indicated that both mRNA and protein levels of *AEBP1* were both significantly reduced after transfection (Figures 3(c) and 3(d)).

### 3.4. *AEBP1* Promotes GBM Cell Proliferation In Vitro

**Figure 4:**
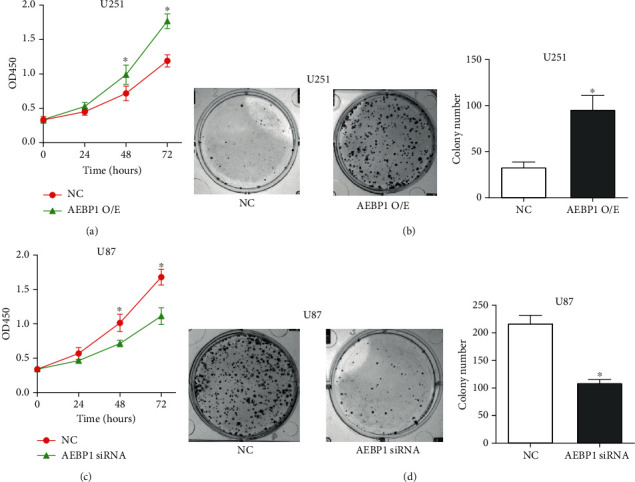
*AEBP1* promoted GBM cell proliferation in vitro. The *AEBP1* overexpression plasmid was transfected in U251 cells, and *AEBP1* siRNA was transfected in U87 cells. Cell proliferation was assessed by the (a, c) CCK8 assay and (b, d) colony formation assay. *N* = 3, ^∗^*P* < 0.05.

Proliferation of *AEBP1*-overexpressing U251 cells and *AEBP1*-silenced U87 cells was assessed by the CCK8 assay and colony formation assay. The results showed that overexpression of *AEBP1* could dramatically promote U251 cell proliferation (Figures 4(a) and 4(b)), while silencing *AEBP1* could inhibit U87 cell proliferation (Figures 4(c) and 4(d)).

### 3.5. *AEBP1* Promotes Migration and Invasion of GBM Cells In Vitro

**Figure 5:**
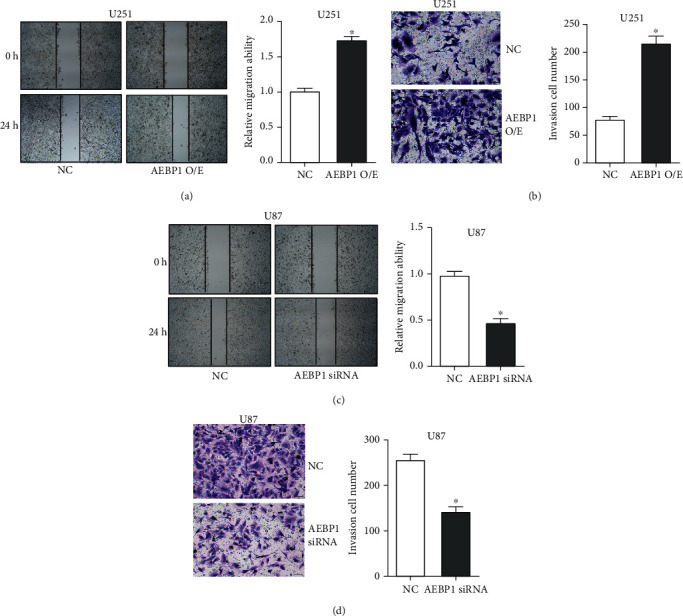
*AEBP1* promoted GBM cell migration and invasion in vitro. The *AEBP1* overexpression plasmid was transfected in U251 cells and *AEBP1* siRNA in U87 cells: (a, c) cell migration was detected by the scratch assay; (b, d) cell invasiveness was assessed by the Transwell assay. *N* = 3, ^∗^*P* < 0.05.

Migration and invasiveness of *AEBP1*-overexpressing U251 cells and *AEBP1*-silenced U87 cells were assessed by the scratch assay and Transwell assay, respectively. The results showed that overexpression of *AEBP1* could dramatically promote U251 cell migration (Figure 5(a)) and invasion (Figure 5(b)), while silencing *AEBP1* could inhibit instead U87 cell migration (Figure 5(c)) and invasion (Figure 5(d)).

### 3.6. *AEBP1* Promotes the Growth of GBM Tumors In Vivo

**Figure 6:**
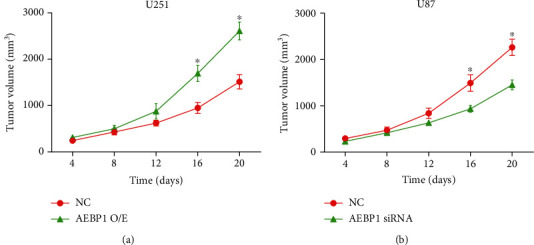
*AEBP1* promoted the growth of GBM tumors in vivo. *N* = 3, ^∗^*P* < 0.05.

U251 cells stably overexpressing *AEBP1* or U87 cells stably silencing *AEBP1* after lentiviral transfection were injected subcutaneously to recapitulate subcutaneous tumor models. By observing the growth rate of subcutaneous tumors, we found that tumor growth was significantly accelerated in the group with *AEBP1* overexpression (Figure 6(a)), while the growth of tumors in the group with *AEBP1* silencing was significantly slowed down (Figure 6(b)).

### 3.7. *AEBP1* Activates the NF-*κ*B Signaling Pathway in GBM Cells

**Figure 7:**
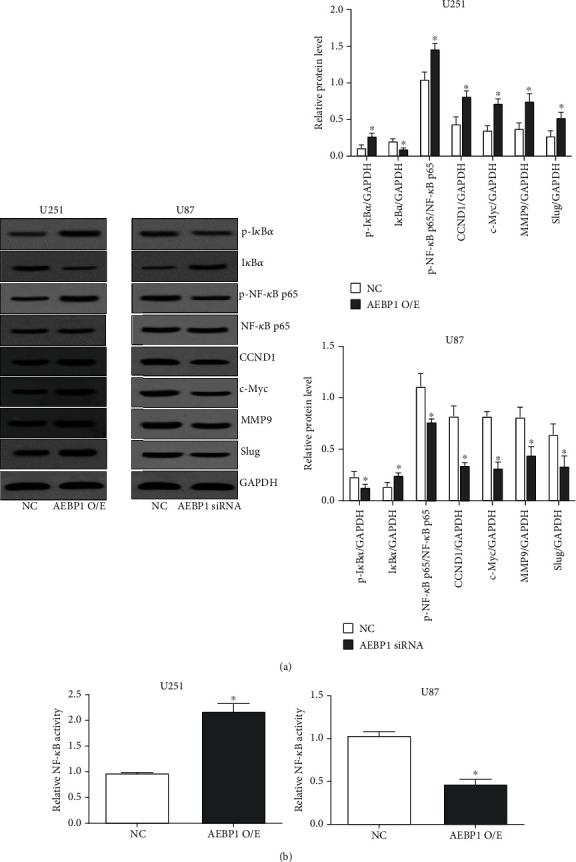
*AEBP1* activated the NF-*κ*B signaling pathway in GBM cells: (a) the expression and phosphorylation levels of proteins related to the classical NF-*κ*B pathway were detected by western blot; (b) a luciferase reporter assay was used to assess the transcriptional activity of NF-*κ*B. *N* = 3, ^∗^*P* < 0.05.

Classical NF-*κ*B pathway engagement was assessed by western blot in *AEBP1*-overexpressing U251 cells and *AEBP1*-silenced U87 cells. The results showed that after *AEBP1* overexpression, the level of phosphorylation of *IκBα* was significantly increased, the I*κ*B*α* level was decreased, and the level of phosphorylation of *NF-κB p65* was significantly increased (Figure 7(a)). Conversely, after silencing of *AEBP1*, the level of phosphorylation of *IκBα* was significantly lowered, the *IκBα* level was elevated, and the level of phosphorylation of *NF-κB p65* was significantly lowered (Figure 7(a)). Furthermore, the luciferase reporter gene assays confirmed that whereas the transcription activity of NF-*κ*B was significantly increased after *AEBP1* overexpression, it was significantly reduced instead after silencing of *AEBP1*. Notably, we found that *AEBP1* could promote the expression of the proliferation-related genes *CCND1* and *c-Myc* and the epithelial-mesenchymal transition- (EMT-) related genes *MMP9* and *Slug* regulated by the NF-*κ*B pathway, while silencing *AEBP1* inhibited instead the expression of these genes (Figure 7(a)).

### 3.8. *AEBP1* Promotes the Expression of *CCND1*, *c-Myc*, *MMP9*, and *Slug* In Vivo

**Figure 8:**
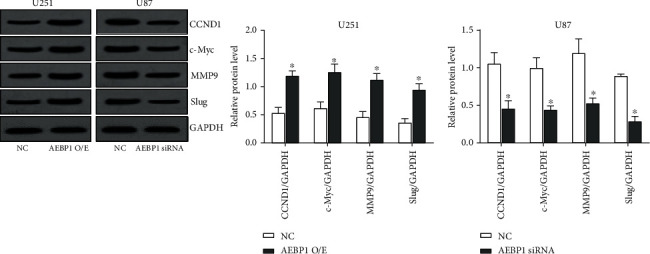
*AEBP1* promotes the expression of *CCND1*, *c-Myc*, *MMP9*, and *Slug* in vivo. Western blot was used to detect the expression levels of *CCND1*, *c-Myc*, *MMP9*, and *Slug* in subcutaneous GBM xenografts formed in nude mice. *N* = 3, ^∗^*P* < 0.05.

We excised subcutaneous tumors formed in nude mice and measured the expression of *CCND1*, *c-Myc*, *MMP9*, and *Slug* by western blot (Figure 8). Consistent with our in vitro analyses, we found that overexpression of *AEBP1* could promote the expression of *CCND1*, *c-Myc*, *MMP9*, and Slug in vivo, while silencing of *AEBP1* could inhibit instead the expression of these genes.

## 4. Discussion

GBM is one of the deadliest diseases of the central nervous system, and the survival rate and life expectancy of GBM patients are bleak [1, 2]. Despite great advances in understanding the genetic basis of gliomas, little progress has been made in exploring the molecular mechanisms underlying their malignant progression [16]. Recent studies have focused on identifying oncogenes or tumor suppressor genes that play an important role in promoting or inhibiting the glioma development and progression [17]. Next-generation sequencing technologies have facilitated the refinement of tumor-related databases, making it easier to discover other key aberrantly expressed genes [18]. In this study, we found that *AEBP1* is highly expressed in GBM through tumor-related databases and bioinformatics analysis, which correlates with a poor prognosis for the patients. This outcome suggests that *AEBP1* may be aberrantly expressed in GBM and is involved in GBM development.

Subsequently, we verified the high expression of *AEBP1* in GBM tissues and cell lines and found that the expression of *AEBP1* was closely related to the tumor size of GBM. This result suggests that *AEBP1* can be used as a biological marker of GBM to indicate the malignant progression of the tumor. Previous studies have reported that high expression of *AEBP1* is also correlated with clinical characteristics of malignant tumors. For example, high expression of *AEBP1* is related to tumor size, histological differentiation, lymph node metastasis, and tumor stage in patients with colon adenocarcinoma [19]; similarly, elevated *AEBP1* expression in gastric cancer is positively correlated with the T stage, N stage (*P* = 0.005), and TNM staging [12]. Therefore, *AEBP1* may have a clinical predictive value in many types of tumors.

Previous studies have focused on the role of *AEBP1* in GBM in relationship with apoptosis [13, 14] as well as other tumorigenic characteristics [5]. In this study, we further investigated the roles of *AEBP1* in GBM progression via a series of in vitro and in vivo analyses. In vitro experiments revealed that *AEBP1* could significantly enhance the proliferation, migration, and invasion ability of GBM cells; in vivo experiments further demonstrated that *AEBP1* was able to contribute to the growth of GBM tumors. Therefore, *AEBP1* promotes tumor progression in GBM through its oncogenic properties, and its tumorigenic role in GBM can be extended to many other tumor types. For example, *AEBP1* in colon cancer promotes cell proliferation, migration, and in vitro tube formation [10]. *AEBP1* in gastric cancer can significantly promote the proliferation, migration, invasion, and epithelial-mesenchymal transition of gastric cancer cells [12]. Thus, *AEBP1* plays a cancer-promoting role in many tissues and may be a potential target for tumor therapy.

In the present study, we found that *AEBP1* in GBM cells could promote the phosphorylation of *IκBα* and downregulate *IκBα* expression, which in turn promoted the phosphorylation of *NF-κB p65* and activated the classical NF-*κ*B signaling pathway. The NF-*κ*B pathway is a key regulator of tumor cell proliferation, apoptosis, angiogenesis, inflammation, metastasis, and drug resistance. Aberrant NF-*κ*B signaling is involved in the pathogenesis of most human malignancies. Consequently, it is now used as an important target for cancer therapy [20–22]. *CCND1* and *c-Myc* are proliferation-related genes [23], and *MMP9* and *Slug* are EMT- (cell migration and invasiveness) related genes downstream of the classical NF-*κ*B pathway [23, 24]. This study showed that *AEBP1* could upregulate the expression of these genes both in vitro and in vivo. Therefore, *AEBP1* may elevate the expression of genes that is involved in proliferation, migration, and invasion by activating the NF-*κ*B signaling pathway, thereby promoting the biological process in GBM. Additionally, previous studies have reported that *AEBP1* activates the NF-*κ*B signaling pathway and promotes tumor progression and drug resistance in colon adenocarcinoma [19], gastric cancer [12], and melanoma [25]. We demonstrated for the first time that *AEBP1* promotes GBM proliferation, migration, and invasion by activating the classical NF-*κ*B pathway.

## 5. Conclusion

Our study confirmed that *AEBP1* is upregulated in GBM and can be used as a valuable biological marker. *AEBP1* promotes GBM cell proliferation, migration, and invasiveness and facilitates tumor growth in vivo by activating the classical NF-*κ*B pathway. This outcome can be used as a potential therapeutic target for the clinical treatment of GBM.

## Figures and Tables

**Table 1:** Relationship between *AEBP1* expression and clinicopathological features in GBM patients.

Pathological factors	*N*	High *AEBP1* expression	Low *AEBP1* expression	*P*
Gender				
Male	27	15	12	
Female	24	11	13	0.488
Age				
<50 years old	20	11	9	
≥50 years old	31	15	16	0.645
IDH1 mutation				
Yes	14	6	8	
No	37	20	17	0.475
Maximum tumor diameter				
<5 cm	23	6	17	
≥5 cm	28	20	8	0.036
Tumor location				
Supratentorial	29	17	12	
Infratentorial	22	9	13	0.210

Statistical analysis was performed using the chi-square test.

## Data Availability

All the data is available with the handwritten notebook documented in our lab.

## Supplementary Materials

Supplementary Materials Supplementary Figure 1: pathological identification of GBM tissue and adjacent tissues: (A) GBM tissue; (B) adjacent tissue.
