# Supplementary material for: AEBP1 Promotes Glioblastoma Progression and Activates the Classical NF-κB Pathway
Source: Behav Neurol. 2020 Nov 6;2020:8890452. doi: 10.1155/2020/8890452 (PMC7665936; doi:10.1155/2020/8890452)
Supplement: Supplementary Materials — Supplementary Figure 1: pathological identification of GBM tissue and adjacent tissues: (A) GBM tissue; (B) adjacent tissue. [file 8890452.f1.docx]

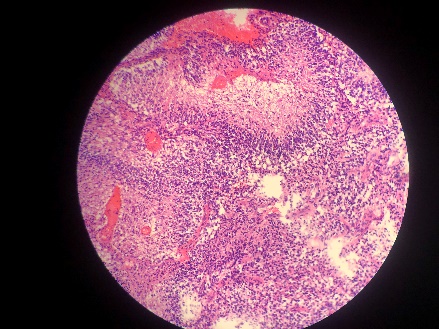

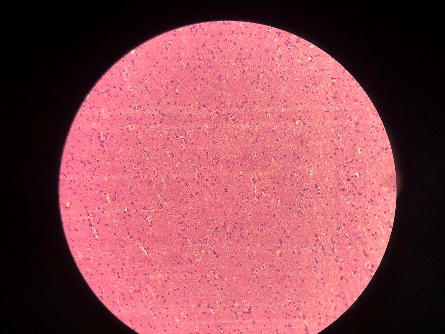


B

A

Supplementary figure 1. Pathological identification of GBM tissue and adjacent tissues. A, GBM tissue; B, adjacent tissue.
